# Mitochondrial-Targeted Curcuminoids: A Strategy to Enhance Bioavailability and Anticancer Efficacy of Curcumin

**DOI:** 10.1371/journal.pone.0089351

**Published:** 2014-03-12

**Authors:** Cheruku Apoorva Reddy, Venkateswarlu Somepalli, Trimurtulu Golakoti, Anantha KoteswaraRao Kanugula, Santosh Karnewar, Karthikraj Rajendiran, Nagarjuna Vasagiri, Sripadi Prabhakar, Periannan Kuppusamy, Srigiridhar Kotamraju, Vijay Kumar Kutala

**Affiliations:** 1 Department of Clinical Pharmacology & Therapeutics, Nizam's Institute of Medical Sciences, Hyderabad, A.P., India; 2 Laila Impex R&D Center, Vijayawada, A.P., India; 3 Centre for Chemical Biology, Indian Institute of Chemical Technology, Hyderabad, A.P., India; 4 National Centre for Mass Spectrometry, Indian Institute of Chemical Technology, Hyderabad, A.P., India; 5 Department of Radiology, Geisel School of Medicine, Dartmouth College, Hanover, New Hampshire, United States of America; Rajiv Gandhi Centre for Biotechnology, India

## Abstract

Although the anti-cancer effects of curcumin has been shown in various cancer cell types, in vitro, pre-clinical and clinical studies showed only a limited efficacy, even at high doses. This is presumably due to low bioavailability in both plasma and tissues, particularly due to poor intracellular accumulation. A variety of methods have been developed to achieve the selective targeting of drugs to cells and mitochondrion. We used a novel approach by conjugation of curcumin to lipophilic triphenylphosphonium (TPP) cation to facilitate delivery of curcumin to mitochondria. TPP is selectively taken up by mitochondria driven by the membrane potential by several hundred folds. In this study, three mitocurcuminoids (mitocurcuminoids-1, 2, and 3) were successfully synthesized by tagging TPP to curcumin at different positions. ESI-MS analysis showed significantly higher uptake of the mitocurcuminoids in mitochondria as compared to curcumin in MCF-7 breast cancer cells. All three mitocurcuminoids exhibited significant cytotoxicity to MCF-7, MDA-MB-231, SKNSH, DU-145, and HeLa cancer cells with minimal effect on normal mammary epithelial cells (MCF-10A). The IC_50_ was much lower for mitocurcuminoids when compared to curcumin. The mitocurcuminoids induced significant ROS generation, a drop in ΔØm, cell-cycle arrest and apoptosis. They inhibited Akt and STAT3 phosphorylation and increased ERK phosphorylation. Mitocurcuminoids also showed upregulation of pro-apoptotic BNIP3 expression. In conclusion, the results of this study indicated that mitocurcuminoids show substantial promise for further development as a potential agent for the treatment of various cancers.

## Introduction

Curcumin has been shown to exhibit anti-inflammatory, antimutagenic, anticancer and antioxidant properties [1], [2]. It has been well established that curcumin inhibits the proliferation and survival of almost all types of tumor cells [3]. Curcumin induces cell-cycle arrest at G1/S or G2/M phases by inhibiting the expressions of cyclin D1 or cdc2/cyclin B, respectively. The major mechanism by which curcumin induces cytotoxicity in tumor cells is by the induction of apoptosis through mitochondrial pathway involving caspase-8, BID cleavage, cytochrome c release, and activation of caspase-3. The proapoptotic members of the Bcl-2 family, such as Bax, are activated, and antiapoptotic genes such as Bcl-XL are inhibited by curcumin [4]. In our earlier studies, we have demonstrated that curcumin and curcumin analogs exhibited significant growth arrest and apoptosis in a number of human cancer cell lines including breast, ovarian, and prostate cancer with no apparent toxicity to noncancerous cells [5]–[7]. The apoptosis induced by curcumin in tumor cells is mediated by multiple signaling pathways that include p53, NF-κB, Akt and JAK-STAT pathways [3].

BNIP3 (Bcl-2/adenovirus E1B 19 kDa-interacting protein3) is a pro-apoptotic member of the Bcl-2 family of apoptotic proteins and it is localized primarily in the mitochondrial outer membrane [8]. Overexpression of BNIP3 leads to the activation of Bax/Bak, opening of the mitochondrial permeability transition pore, and cell death [9]. BNIP3 functions as a mitochondrial sensor of oxidative stress, where an increase in ROS induces the homodimerization and activation of BNIP3 *via* a conserved cysteine residue in the NH_2_ terminus [10]. Recent studies suggest that curcumin plays a role in cancer epigenetics through its interaction with histone deactylases, histone acetyl transferases, DNA methyl transferases and micro RNAs [11].

Although the curcumin has been shown to exhibit anticancer efficacy in various cancer cell types *in vitro*, its efficacy in pre-clinical and clinical studies is limited, even at high doses. This is presumably due to its poor water-solubility and short biological half-life, resulting in low bioavailability in both plasma and tissues [12]. To overcome these limitations, several approaches, including combination of curcumin with adjuvants (e.g., piperine), and the development of delivery vehicles consisting of liposomes, nanoparticles, phospholipid formulations and synthetic curcumin analogs have been tested *in vitro* [12].

Mitochondria of cancer cells and transformed cells have significantly higher transmembrane potential than normal cells [13]. This biological property has been used as a basis to develop compounds that may preferentially accumulate within the mitochondria of cancer cells for selective targeting of drugs to mitochondrion. One such approach to deliver to mitochondria is by coupling of curcumin to lipophilic triphenylphosphonium (TPP) cation. TPP is selectively taken up by mitochondria because of its high negative membrane potential (140–170 mV, negative side) [14]. Based on this observation, the TPP cation has been used as a targeting moiety for delivery of agents such as spin traps, fluorescence dyes, and antioxidants to isolated mitochondria, as well as the mitochondria of intact cells and whole organisms [15]. The aim of this study was (i) to synthesize mitochondrially-targeted curcuminoids, termed as mitocurcuminoids, to enhance the intracellular/mitochondrial uptake of curcumin, (ii) to study the anticancer efficacy of these compounds and (iii) to derive mechanistic insights into the action of mitocurcuminoids. Since ROS generation and alterations in mitochondrial membrane potential may influence BNIP3 expression, the effect of mitocurcuminoid on the expression of BNIP3 and the epigenetic alterations influencing BNIP3 expression by mitocurcuminoid were also studied.

## Materials and Methods

Curcumin (1,7-bis[4-hydroxy-3-methoxyphenyl]-1,6-heptadiene-3,5-dione) and triphenyl-phosphine were procured from Sigma-Aldrich (USA). Synthesis of mitocurcuminoids-1, 2 and 3 (now referred as mitocur-1, mitocur-2 or mitocur-3) and characterization are provided in File S1. Stock solutions of 2–100 mM curcumin and mitocurcuminoids were prepared in DMSO and stored at −20°C.

### Cancer Cell Cultures

MCF-7 human breast cancer cell line was used for most of the studies reported in this work. The other cancer cell lines used were MDA-MB-231, SK-N-SH, DU-145, and HeLa. All cell lines were obtained from ATCC. MCF-7 and MDA-MB-231 cells were grown in DMEM supplemented with 10% FBS, 2% sodium pyruvate, non-essential amino acids (2 mM), penicillin (100 units/ml), streptomycin (100 µg/ml), and glutamine (4 mM). SK-N-SH, DU-145, and HeLa cells were grown in MEM. Normal mammary epithelial cells (MCF-10A) were grown in MEBM supplemented with BPE, hEGF, insulin and hydrocortisone. Cells were grown to 70% confluence at 37°C in a humidified atmosphere of 5% CO_2_ and 95% air.

### Cell Counting

Untreated and mitocucuminoid-1, 2, 3 or curcumin-treated cells were counted using a Countess automated cell counter (Invitrogen).

### Isolation of cytosolic and mitochondrial fractions from MCF-7 cells

MCF-7 cells were grown in 90-mm dishes, and then treated with or without 10 µM of Mitocur-1, 2, 3 or curcumin for 6 h. After the treatment, cells were washed thrice with PBS. The isolation of mitochondrial and cytosolic extracts was carried out using a commercially available ProteoExtract Cytosol/Mitochondria Fractionation Kit (Merck, USA) according to manufacturer's instructions.

### Detection and quantification of Mitocur-1, 2, and 3 in MCF-7 cell extracts by Mass Spectrometry

The electrospray ionization (ESI)-MS (positive mode) measurements were performed using a quadrupole time-of-flight mass spectrometer (QSTAR XL, Applied Biosystems/MDS Sciex, Foster City, CA, USA). The data acquisition was under the control of Analyst QS software (Applied Biosystems). For the CID (collision-induced dissociation) experiments, the precursor ions were selected using the quadrupole analyzer and the product ions were analyzed using the TOF analyzer.

The mitochondrial/cytosolic extracts (50 µl) were diluted with 50 µl of methanol, and introduced into the ESI source by flow injection (10 µl loop) using methanol as the mobile phase at the flow rate of 30 µl/min. Stock solutions (1 mM) of all the standards were made in methanol:water (75∶25, v/v), and a drop of DMSO, if required, was used to dissolve the compound. For spiking experiments, appropriate volumes of standard solutions (1–50 µM) were added to the mitochondrial/cytosolic extracts of untreated cells.

### Sulforhodamine B (SRB) assay

Cells were grown in 24-well plates and treated with various concentrations (0.1–50 µM) of Mitocur-1, 2, 3 or curcumin and also with triphenylphosphonium (TPP) for a period of 24 h. The cell viability was assessed by using SRB assay [16].

### Detection of intracellular superoxide

MCF-7 cells were treated with Mitocur-1 (10 µM) or curcumin (50 µM) for 4 h with and without antioxidant, N-acetyl cysteine (NAC, 4 mM). After the treatment, cells were incubated with 10 µM DHE, for 30 min. Cells were then washed with PBS and fluorescence images were immediately captured with a Nikon Eclipse TE2000-U camera system using excitation/emission at 488/585 nm. Images were analyzed using MetaMorph image analysis software. Further the ROS generation by Mitocur-1 or curcumin in MCF-7 cells was determined by EPR spectroscopy (for procedure see File S1).

### Cell-cycle analysis by Flow cytometry

For DNA content analysis, MCF-7 cells were treated with Mitocur-1 (10 µM) for 24 h. Cells were harvested and centrifuged for 5 min at 300 g, fixed by the gradual addition of ice cold 70% ethanol and washed with PBS. Cells were then treated with RNase (10 µg/ml) for 30 min at 37°C, washed twice with PBS, and resuspended and stained with 1 ml of propidium iodide (69 µM) containing 38 mM sodium citrate for 30 min at room temperature [17]. The cell cycle phase distribution was determined and the percentage of cells in each phase of the cell cycle was analyzed using ModFit LT software (BectonDickinson).

### Detection of mitochondrial transmembrane potential

Mitochondrial potential was assessed using the fluorescent potentiometric dye JC-1 (5,5′,6,6′-tetrachloro-1,1′, 3, 3′-tetraethylbenzimidazolcarbocyanine iodide) (Molecular Probes, Eugene, OR). In healthy cells, JC-1 forms J-aggregates that display a strong red fluorescence with excitation of 560 nm and emission wavelength at 595 nm. In apoptotic or unhealthy cells, JC-1 exists as monomers that display a strong green fluorescence with excitation and emission at 485 nm and 535 nm, respectively. MCF-7 cells were treated with Mitocurc-1, 2, or 3 for 4 h. At the end of the treatments, cells were washed with DPBS, incubated with JC-1 dye (5 µg/ml) for 20 min and observed under the fluorescence microscope.

### Caspase 3-like and caspase 8 activity

MCF-7 cells were treated with Mitocur-1 (5 µM) or curcumin (5 µM) for 24 h. Cells were then washed twice in cold DPBS and lysed in buffer containing 10-mM Tris-HCl, 10-mM NaH_2_PO_4_/Na_2_HPO_4_ pH.7.5, 130-mM NaCl, 1% Triton, and 10-mM sodium pyrophosphate. Cell lysates were incubated with caspase 3 fluorogenic substrate, N-acetyl-Asp-Glu-Val-Asp-7-amido-4-methylcoumarin, or caspase 8 fluorogenic substrate, N-acetyl-Ileu-Glu-Thr-Asp-7 amido-4-methylcoumarin (Sigma) at 37°C for 1 h. The 7-amido-4-methyl-coumarin liberated from the substrate was measured using a fluorescence plate reader (Tecan M-200) with λ_ex_ = 380 nm and λ_em_ = 460 nm.

### Western blot analysis

After the treatments with either curcumin or with Mitocur-1 for 24 h, MCF-7 cells were lysed in RIPA buffer containing protease and phosphatase-inhibitor cocktail and centrifuged at 10,000 rpm for 20 min at 4°C and supernatant was collected and proteins were resolved by SDS–PAGE, blotted onto a nitrocellulose membrane and incubated with Bcl2, Bax, caspase-7, PARP, cyclin B1, cyclin A, cyclin D1, pSTAT(Tyr-705), STAT, pAkt (Thr-308), Akt, pERK1/2, ERK antibodies in separate experiments. The detection was carried out with either HRP-labeled sheep anti-mouse IgG or HRP-labeled donkey anti-rabbit IgG (Amersham) using an enhanced chemiluminescence detection system (ECL Advanced Kit, GE Health care).

### BNIP3 expression by RT-PCR

Total RNA was isolated from MCF-7 using TRIzol method according to manufacturer's instructions (Invitrogen). Quantitative RT-PCR was performed using gene specific forward (FP) and reverse primers (RP) of BNIP3(FP:5′CCACCTCGCTCGCAGACACCAC3′, RP:5′GAGAGCAGCAGAGATGGAAGGAAAAC3′) respectively.

### Statistical analysis

Data are expressed as mean ± SD. Comparisons among the groups were performed using Student- t test. The significance level was set at p<0.01. IC_50_ was measured from logarithmic regression equation of dose versus percentage of inhibition cell proliferation.

## Results

**Figure 1:**
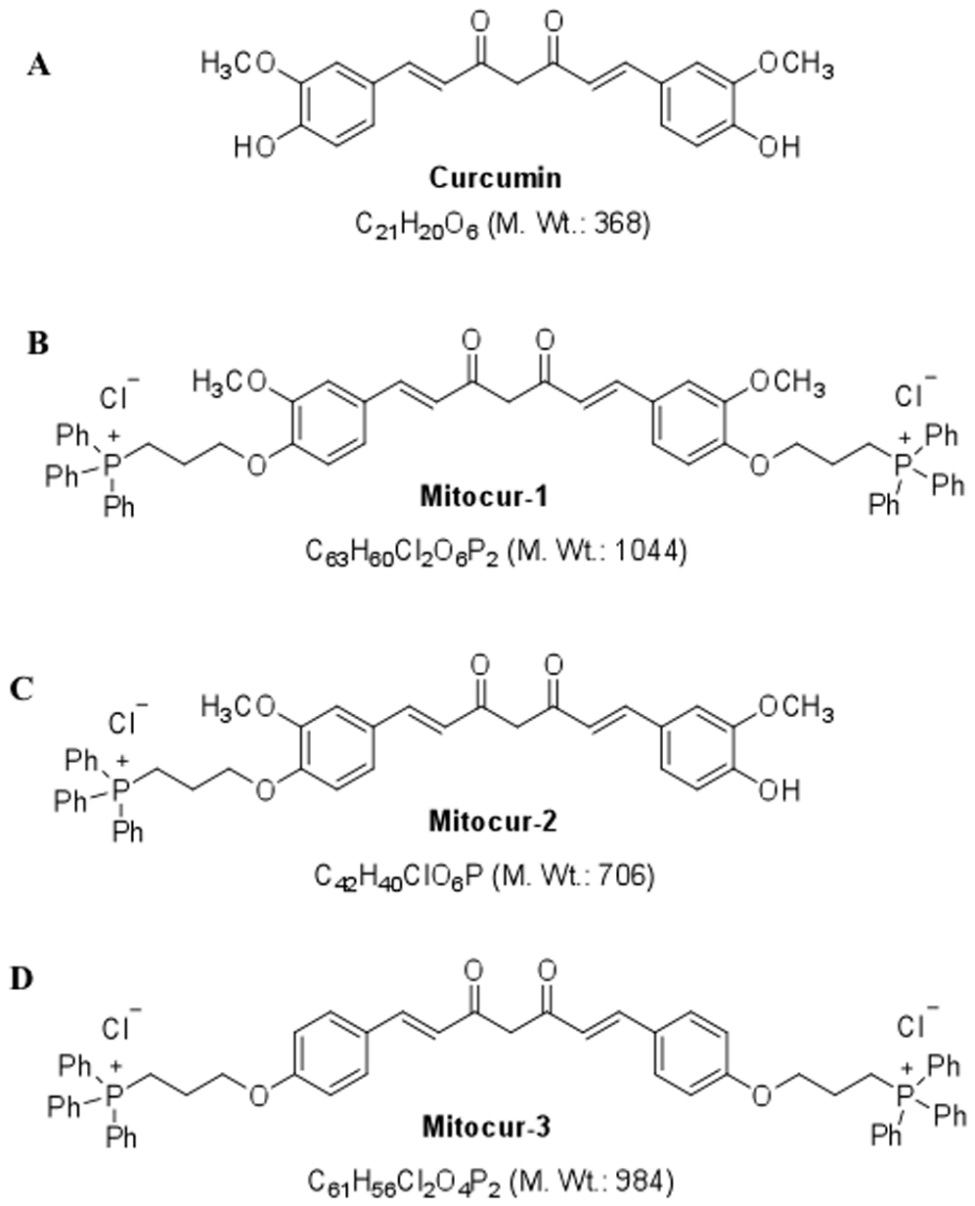
Chemical structure of the mitochondria-targeted curcuminoids used in this study. (**A**) Curcumin; (**B**) Mitocur-1; (**C**) Mitocur-2; (**D**) Mitocur-3. The synthetic curcuminoids (Mitocur-1,2,3) have a triphenylphosphonium cation linked to the hydroxyl group(s) of curcumin via a three-carbon propyl chain. Mitocur-3 is similar to Mitocur-1, but is devoid of methoxy substitution on the aryl groups.

The synthesis, structural analysis of Mitocur-1, 2, and 3 and their cellular uptake (Fig. S1, S2, S3, S4, S5, S6, S7) in MCF-7 cells are shown in supporting information. Fig. 1 shows the structures of Mitocur-1, 2, and 3. HRMS data of these compounds is shown in Table S1 in File S1.

### Quantification of Mitocur-1, 2 or 3 in mitochondrial/cytosolic fractions of MCF-7 cells

Standard calibration curve (Fig. S4, S5, S6, S7) for each compound (Mitocur-1, 2, or 3) was constructed using the ion counts of the respective peaks in the spectra of spiked samples (average of triplicate data). The quantities of the mitocurcuminoids from the extracts of treated cells were obtained using the ion count of the particular ion and the linear regression equation of the calibration curve (Table 1 and Fig. S4, S5, S6, S7). Table 1 clearly shows that the accumulation of mitocurcuminoids in mitochondria more than in cytosol. Also, Mitocur-1 and 3 accumulated more in the mitochondrial fraction of MCF-7 cells as compared to Mitocur-2 that could be due to the presence of two TPP tags at either side of the curcumin molecule (Table 1).

**Table 1:** Cellular uptake of curcumin and mitocurcuminoids-1, 2, and 3.

Compound	Mitochondrial fraction (n mol/mg protein)	Cytosolic fraction (n mol/mg protein)
Curcumin	ND	89±10
Mitocur-1	1680±106	164±32
Mitocur-2	670±98	184±38
Mitocur-3	1474±162	178±10

MCF-7 cells were treated with either curcumin (10 µM) or Mitocur-1, 2, 3 (10 µM) respectively for a period of 6 h. Mitochondrial and cytosolic fractions were isolated as described in Methods section. Values represented are Mean±SD of three independent experiments.

### Mitocurcuminoids (1, 2, or 3) are significantly toxic to MCF-7, MDA-MB-231, DU-145, HeLa and SKNSH cells

The cytotoxic effects of mitocurcuminoids were determined and compared with that of free curcumin and TPP in MCF-7, MDA-MB-231, HeLa, DU-145, and SK-N-SH cells. The IC_50_ values are presented in Table 2. Among the different cancer cell lines tested, it was observed that MCF-7 cells were the most susceptible to mitocurcuminoid-induced cell death. Of the mitocurcuminoids, Mitocur-1 was found to be more potent and for this reason, all the subsequent studies to understand the mechanistic aspects of mitocurcuminoid-induced cancer cell death were carried out in MCF-7 cells. Nevertheless, compared to free curcumin, all three mitocurcuminoids showed significant cytotoxicity to all the cancer cell lines tested in this study (Table 2).

**Table 2:** Curcumin and Mitocur-1, 2 and 3 induce cell death in various cancer cells by SRB Assay.

Compound	MCF-7 IC_50_ (µM)	MDA-MB-231 IC_50_ (µM)	HeLa IC_50_ (µM)	DU-145 IC_50_ (µM)	SKNSH IC_50_ (µM)
Curcumin	40.32	37.87	48.07	50	38.46
Mitocur-1	2.31	5.1	4.46	5.10	5.12
Mitocur-2	5.31	8.33	7.69	8.62	8.19
Mitocur-3	3.84	6.75	4.38	6.75	6.28

MCF-7, MDA-MB-231, HeLa, DU-145, and SKNSH cells were incubated with curcumin, mitocurcuminoids-1, 2, or 3 at a dose ranging from 0.1–50 µM for a period of 24 h. At the end of the treatments, cell death was assayed by SRB assay as described in Methods section. Results are expressed as IC_50_ (µM). Values represent data in triplicates from at least three independent experiments.

The cytotoxic effects of mitocurcuminoids were also studied in normal mammary epithelial cells (MCF-10A). The results (Fig. S8) shows that there was no significant effect of mitocurcuminoids on MCF-10A cells. Separate experiments were performed on the cytotoxic effect of TPP alone on MCF-7 breast cancer cells. TPP was tested at different concentrations (1, 5 10 µM) for 24 h and the results showed no toxicity of TPP alone (Fig. S9)

### Mitocurcuminoids induces ROS generation in MCF-7 cells

**Figure 2:**
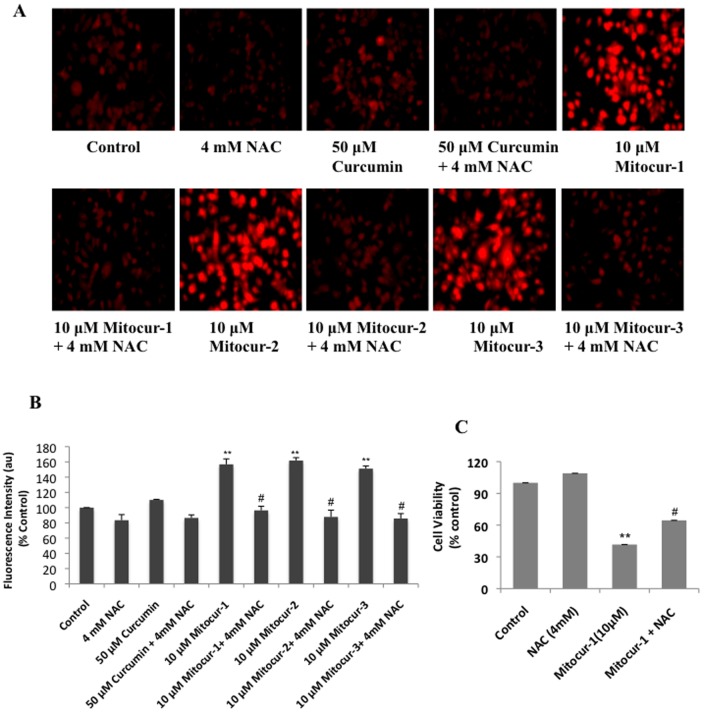
Mitocur-1, 2 and 3 induces superoxide generation in MCF-7 cells. (**A**) Ethidium fluorescence of MCF-7 cells treated with curcumin (50 µM) and mitocur-1, 2, 3 (10 µM) for 4 h. All experiments were performed with and without NAC (4 mM). Representative fluorescence images are shown. (**B**) shows quantification of fluorescence intensities shown in A. **, significantly different compared to control (P<0.01) and #, significantly different compared to mitocurcuminoid-1, 2 and 3 (P<0.01). (**C**) cells were treated with or without 10 µM mitocurcuminoid-1 and 4 mM NAC for 24 h. Results are expressed percentage of viable cells compared to control. Data represented is from at least three independent experiments. **, significantly different compared to control (P<0.01) and #, significantly different compared to NAC (P<0.01).

MCF-7 cells treated with the mitocurcuminoids (at 10 µM for 4 h) showed significant increase in ethidene fluorescence as an indicator of superoxide generation (Fig. 2A–B). This increase in ethidine fluorescence was significantly abrogated in cells pretreated with N-acetylcysteine (NAC, 4 mM). The inhibition of ROS generation by NAC was also evident from the cell-viability measurements, which showed significant inhibition of the cytotoxic effects induced by Mitocur-1 (Fig. 2C).

### Mitocur-1 generates superoxide in MCF-7 cells

**Figure 3:**
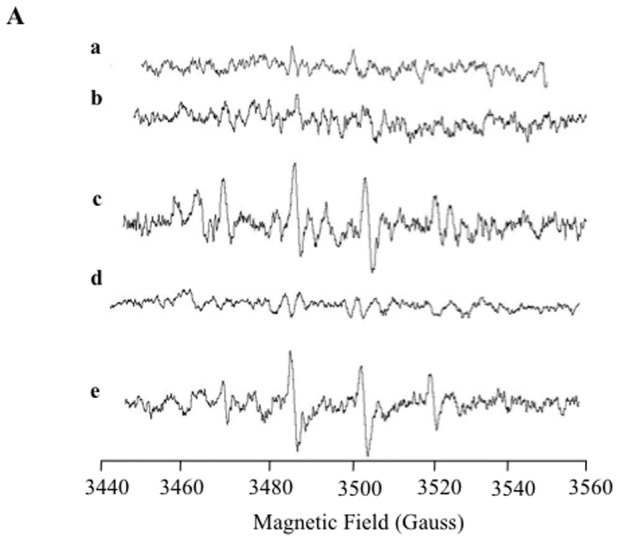
Superoxide generation in MCF-7 cells treated with mitocur-1 detected by EPR spectroscopy using DMPO spin trap. (**A**) Cells alone; (**B**) Cells+curcumin (10 µM); (**C**) Cells+mitocur-1Mitocur- (10 µM); (**D**) Cells+mMitocur-1 (10 µM)+MnTBAP (10 µM); (**E**) xanthine+xanthine oxidase. Microwave frequency, 9.786 GHz; microwave power, 10 mW; modulation amplitude, 1 G; scan time, 30 s; no. of scans, 10.

The superoxide generation by Mitocur-1 in MCF-7 cells was further confirmed by EPR spectroscopy using DMPO spin-trap agent. The EPR spectrum of MCF-7 cells treated with Mitocur-1 showed the presence of DMPO-OH adduct (Fig. 3). The origin of the DMPO-OH adduct was confirmed to be superoxide using Mn-TBAP, an intracellular superoxide scavenger. There was no adduct formation in cells treated with curcumin at the tested concentration.

### Mitocur-1 induces loss of mitochondrial membrane potential in MCF-7 cells

**Figure 4:**
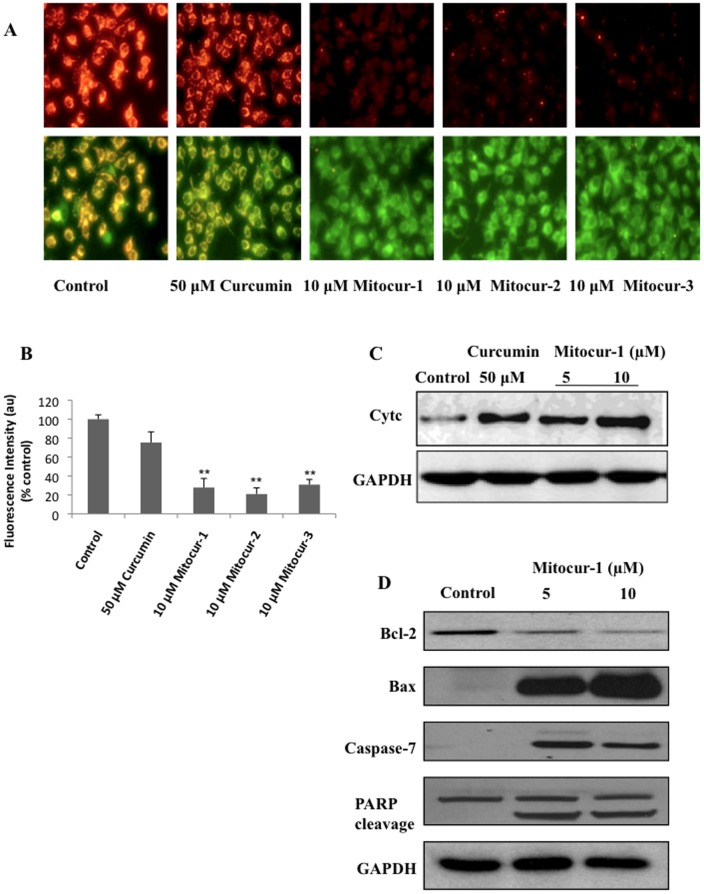
Effect of mitocurcuminoids and curcumin on mitochondrial membrane potential and apoptotic markers. (**A**) Cells were treated with 10 µM Mitocur-1, 2, 3 or 50 µM curcumin for 4 h. Then washed with PBS and incubated with JC-1 dye (5 µg/ml) for 20 min to measure the loss of mitochondrial membrane potential. Fluorescence images were captured in both FITC and rhodamine filters and images showing J-aggregates are represented. (**B**) shows quantification of images (J-aggregates) shown in A. (**C**) Mitochondria and cytosolic fractions were isolated using ProteoExtract Cytosol/Mitochondria Fractionation Kit and cytochrome c levels were measured by Western blot analysis. (**D**) MCF-7 cells were treated with Mitocur-1 (5 and 10 µM) for 24 h. Total protein was resolved by SDS-PAGE electrophoresis and Western blot analysis was performed using respective antibodies for Bcl2, Bax, caspase-7 and PARP. **, significantly different compared to control (p<0.01).

MCF-7 cells treated with Mitocur-1, 2, or 3 showed a substantial reduction in the ratio of red to green fluorescence (Fig. 4A). This indicates a drop in Δ*Ψ*_m_. The untargeted curcumin (50 µM) also did cause a marginal loss of mitochondrial membrane potential. The quantification of J-aggregate fluorescence intensities of the same are shown in Fig. 4B. In tune with the loss of mitochondrial membrane potential, both curcumin at 50 µM and mitocurcuminoid-1 at 5 and 10 µM appreciably increased the release of cytochrome c into the cytosol (Fig. 4C). Mitocur-1 treatment also significantly upregulated Bax and downregulated Bcl-2 expressions (Fig. 4D), which are proapoptotic and antiapoptotic proteins of Bcl family respectively. In line with the above results, MCF-7 cells treated with Mitocur-1 also resulted in the cleaved caspase-7 and degradation of PARP (Fig. 4D).

### Mitocur-1 induces cell cycle arrest and causes cell death by apoptosis pathway in MCF-7 cells

**Figure 5:**
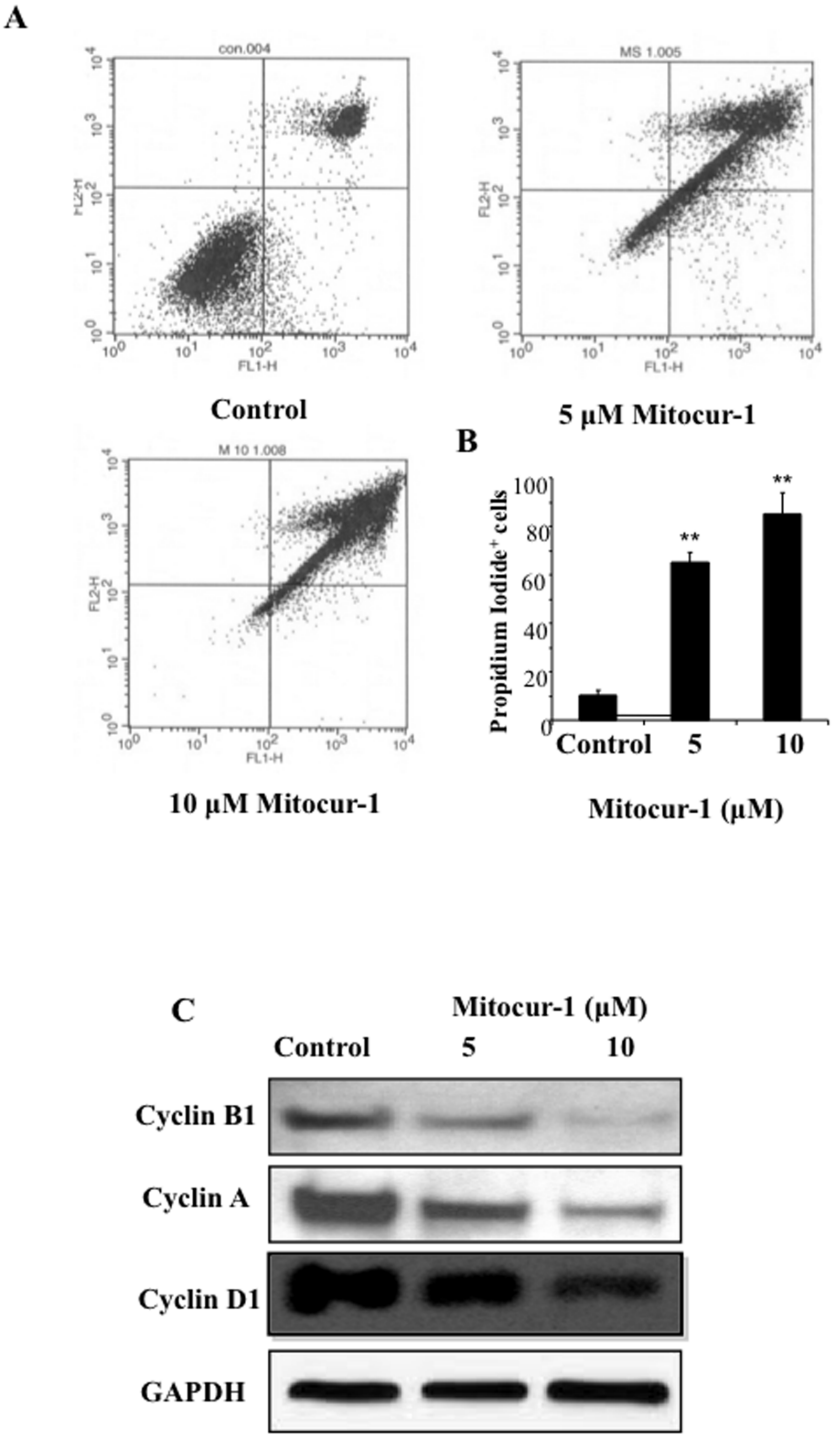
Modulation of cell cycle progression by Mitocur-1. MCF-7 cells were treated with Mitocur-1 (5 and 10 µM) for a period of 24 h. (**A**) shows the flow cytometry profiles of (PI)- stained cells of control, and Mitocur-1 (5 and 10 µM) treatment as described in Methods. (**B**) Quantitative cell cycle (DNA content) distribution (% of total) in the control and treatment groups. (**C**) MCF-7 cells were treated with Mitocur-1 (5 and 10 µM) for 24 h and subjected to Western blot analysis. Representative immunoblot images of cyclin A, cyclin B1 and cyclin D1 are shown. Values are expressed Mean ± SD; (n = 4). **, significantly different from control (P<0.01).

Among the three mitocurcuminoids studied, Mitocur-1 showed significantly higher toxicity than the other two compounds. To see whether the growth inhibition of MCF-7 cells by Mitocur-1 was caused by cell-cycle arrest, we performed flow-cytometry analysis. Cells were treated with Mitocur-1 for 24 h, fixed; and cell-cycle populations were determined by flow cytometry (5A). The results showed that cell population in the G2-M and sub-G1 phases were significantly higher in the treatment group when compared to the untreated control group (Fig. 5B). Mitocur-1 significantly down regulated the cell-cycle regulatory proteins such as, Cyclin A, B1, and, D1 as determined by Western-blot analysis (Fig. 5C). These results indicated that Mitocur-1 modulates both G1/S and G2/M cell-cycle proteins. To determine whether the Mitocur-1–induced cell-cycle arrest led to apoptosis, caspase-3 and caspase-8 enzyme activities were measured. It was observed that caspase-3 activity was increased by 20-fold and caspase-8 by 4.5-fold in Mitocur-1 treated cells as compared to untreated conditions (Table 3). Untargeted curcumin also marginally induced both the caspase activities.

**Table 3:** Mitocur-1 increase caspase 3-like and caspase-8 activities in MCF-7 cells.

Sample	Caspase 3-like activity (% control)	Caspase-8 activity (% control)
Control	100	100
Curcumin (5 µM)	190±6.69	170±6.74
Mitocur-1 (5 µM)	1921±6.83	479±6.50

MCF-7 cells were treated with either curcumin or Mitocur-1 for 24 h and caspase 3-like and caspase-8 activities were measured by using respective substrates as mentioned in Methods. The fluorescence intensity was normalized to mg protein and the values are expressed as % control.

### Mitocur-1 inhibits the STAT3, Akt and ERK pathways

**Figure 6:**
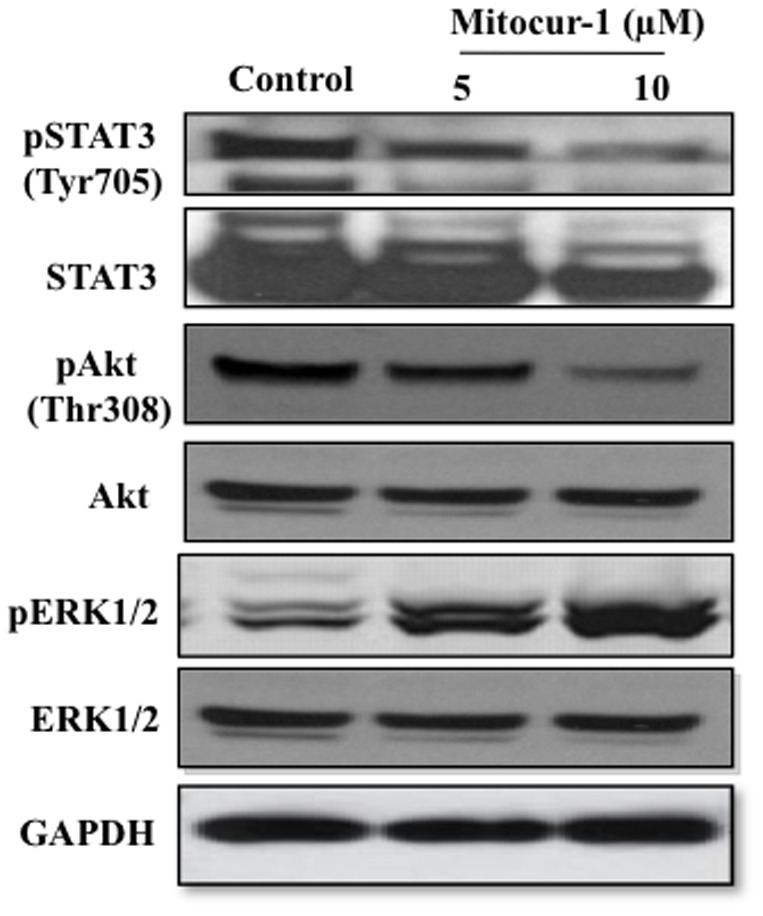
Mitocur-1 causes inhibition of Akt and STAT3 signaling and increase the ERK1/2 phosphorylation. MCF-7 cells were treated with Mitocur-1 (5 or 10 µM) for 16 h and subjected to Western blot analysis as described in Methods. Representative immunoblot images of STAT3, phosphorylated STAT3 (Tyr705), Akt, phospho-Akt (Thr-308), ERK1/2 and phospho- ERK1/2 are shown.

Further, we have investigated whether mitocur-1–induced cell death of MCF-7 cells is mediated by alterations in Akt (Thr-308), STAT3 (Tyr-703) and ERK1/2 (P42/44, Thr202/Tyr 204) phosphorylation statuses. It was found that STAT3 and Akt phosphorylations were decreased but whereas ERK phosphorylation increased significantly in MCF-7 cells treated with Mitocur-1 (10 µM) for a period of 24 h (Fig. 6). The observed results with decreased phosphorylation of STAT3 are in line with the altered expressions of some of the known downstream targets of STAT3 including Bcl2 and Bax as shown in Fig. 6.

### Mitocur-1 regulates BNIP3 expression possibly *via* altering DNMT

**Figure 7:**
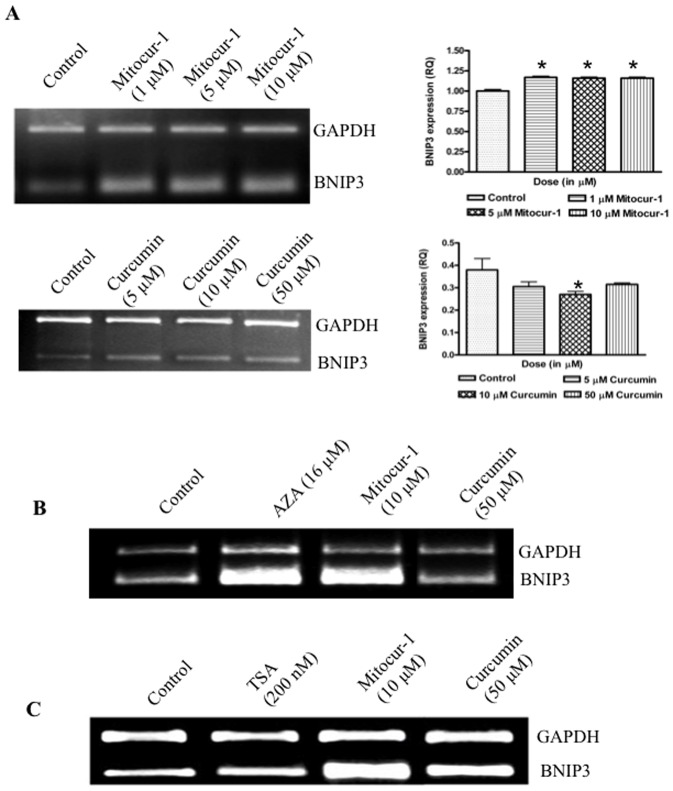
Curcumin and Mitocur-1 presumably regulates BNIP3 expression by modulating DNMT1 but not through HDAC's. (**A**) MCF-7 cells were treated with various concentrations of either mitocurcuminoid-1 (1–10 µM) or curcumin (5–50 µM) for a period of 24 h. Total RNA was extracted by TRIzol method and RT-PCR was performed using gene specific primers of BNIP3. (**B**) and (**C**) are same as A, except that cells were treated with AZA, (24 µM) or TSA (200 nM). Relative quantifications of BNIP3 expressions were presented in bar graphs. *, significantly different compared to control (p<0.01).

Mitocur-1 at sub-micromolar concentrations (5–10 µM) induced BNIP3 expression in MCF-7 cells treated for 24 h. Compared to baseline expression of BNIP3 in MCF-7 cells, treatment of Mitocur-1, showed a significant increase in the BNIP3 expression (Fig. 7A). To study the role of DNA methylation and histone deacetylation on BNIP3 expression, specific inhibitors such as 5-Aza-2′deoxycytidine (AZA), a specific inhibitor of DNA methyltransferase and trichostatin A (TSA), an inhibitor of class 1 and II of histone deacetylases, were used. Treatment of MCF-7 cells with AZA showed an increased expression of BNIP3, suggesting a role for DNA methylation in influencing BNIP3 expression (Fig. 7B). The role of histone acetylation in controlling BNIP3 expression was ruled out by treating the cells with TSA, which did not alter the BNIP3 (Fig. 7C)

## Discussion and Conclusion

In the current study, mitochondrially-targeted mitocurcuminoid-1, 2, and 3 were synthesized by covalently coupling curcumin to lipophilic TPP cation and structures were confirmed by ESI-MS and HRMS. Mitocur-1 and 3 were synthesized by tagging the curcuminoid with two TPP moieties with the only difference being the absence of a methoxy group in Mitocur-3. This was done to see if the presence or absence of a methoxy group will affect the uptake of the compound into the mitochondria. However, it was found that the methoxy substitution did not affect the uptake. When compared to curcumin, Mitocur-1, 2, and 3 accumulated more effectively in the mitochondria of MCF-7 cells. Of the three compounds, Mitocur-1 and 3 accumulated most significantly in the mitochondria of MCF-7 cells as compared to Mitocur-2. The reason for the increased uptake of Mitocur-1 and 3 into the mitochondria could be due to the presence of two TPP moieties in these molecules and hence more anti-proliferative effects in all the cancer cell lines tested when compared to Mitocur-2 that has only one TPP conjugation. Our results also indicate that the absence of OH group in the Mitocur-1 or 3 does not affect their uptake and cytotoxicity towards cancer cells. In a recent study, it was observed that vitamin E analogs like mito-chromanols possessing phenolic hydroxyl group and acetylated ester analog of mito-chromonals lacking a free OH group showed equal potency in breast cancer cells [18], indicating that presence of free OH group is not critical in anticancer efficacy as observed in our study. Further, we tested the effect of mitocurcuminoids on normal epithelial cells, MCF-10A and found no toxicity. The mechanism of anticancer action of Mitocur-1 was observed to be by multiple pathways; increased generation of ROS, increased oxygen consumption (data not shown) measured by using paramagnetic oxygen sensing probe, LiNc-BuO by EPR spectroscopy [19], modulation of proteins involved in G2/M cycle arrest, downregulation of antiapoptotic signals and inhibition of Akt and STAT3 phosphorylation, and increased ERK phoshorylation. The observed mechanism of action of the mitocurcuminoids is similar to that of free curcumin which was demonstrated by us [6] and others [20], indicating that conjugation of TPP does not interfere with the anticancer properties of curcumin. TPP alone showed no toxicity to MCF-7 breast cancer cells. Furthermore, this study, for the first time, demonstrated that Mitocur-1 induced apoptosis, may also in part, involve the upregulation of pro-apoptotic BNIP3, possibly by modulating the regulation of DNA methyl transferase activities.

Several mitochondria-targeted drugs and antioxidants (Mito-Q, Mito-CP, Mito-vitamin E, Mito-peroxidase, and Mito-PBN) have been successfully synthesized and tested [21]–[24]. Studies using *in vivo* animal models with oral administration of lipophilic triphenylphosphonium cations demonstrated significant uptake in liver, heart, and muscle [25]. A recent study reported that Mito-Q was 30-fold more cytotoxic to breast cancer cells than to healthy mammary cells [26]. Similarly, TPP-conjugated resveratrol showed significant accumulation in mitochondria of cancer cells and showed toxicity to cancer cells [27]. The present study demonstrated that Mitocur-1 inhibited the MCF-7 breast cancer cell proliferation by inducing G1/S and G2/M phase of the cell-cycle arrest leading to apoptotic cell death. This was accompanied by the downregulation of both cyclin-A and cyclin B1. Cyclin D1 is overexpressed in many cancers, including breast, esophagus, head and neck, and prostate cancer [28]. Previous studies have shown that curcumin blocks the proliferation of various cancer cells by downregulating the expression of the cyclin D1 protein [29], [30].

In the current study, activation of caspase-8, caspase-7, caspase 3-like activities and cleavage of PARP was observed in MCF-7 cells treated with Mitocur-1 in inducing apoptosis and these results are in agreement with earlier published reports on anti-cancer effects of curcumin [30], [31]. Recent work also showed that curcumin inhibits growth and survival of human head and neck squamous cell carcinoma cells *via* modulation of NF-kB signaling [32]. It is well established that an increase in ROS levels, drop in ΔØ_m_ may activate apoptosis through caspase-3 activation and cytochrome c release. There was an increase in translocation of mitochondrial cytochrome c into the cytosol in cells treated with Mitocur-1 suggesting that TPP-tagged curcuminoids induce mitochondria-dependent pathway of apoptosis.

Akt plays critical roles in mammalian cell-survival signaling and is active in various cancers. The present study showed that mitocurcuminoid dose dependently decreased Akt phosphorylation (Thr-308) in MCF-7 cells. These results indicate a possibility that mitocurcuminoids activate protein phosphatase 2A (PP2A) activity that in turn enhances the dephosphorylation of Akt at Thr-308 as shown previously [33].

STAT3 is also known to protect cells from apoptosis through the upregulation of Bcl-xL, Bcl-2, and survivin. Elevated STAT3 activity has been detected in head and neck squamous cell carcinoma, leukemia, lymphoma and multiple myeloma [34], [35]. In the current study, Mitocur-1 inhibited the STAT3 phosphorylation and downstream targets such as Bcl2 and Bax in MCF-cells. Parent curcumin has been shown to reduce the STAT3 phosphorylation in a variety of cancer cells, however at relatively higher concentrations [34], [35].

In the current study, we observed that Mitocur-1 activates ERK1/2 phosphorylation in MCF-7 cells. Earlier, it was shown that curcumin inhibits the Akt/mTOR/p70S6K pathway and activated the ERK1/2 pathway simultaneously [36]. Curcumin can also act as potent radiosensitizer and cause a significant generation of ROS that further lead to the activation of ERK [36]. The increase in ROS by Mitocur-1, as observed in this study, may have lead to activation of ERK1/2 phosphorylation and subsequently the induction of pro-apoptotic pathway.

BNIP3, a pro-apoptotic member of the Bcl-2 family, is being explored in relation to apoptosis and cancer cell death. The increase in BNIP3 by mitocurcuminoids-1 may be presumably due to increase in oxidative stress and a drop in ΔØ_m_. Expression of BNIP3 is associated with the mitochondrial permeability transition (MPT). In a recent study, we have demonstrated a linear relationship between oxidative DNA damage marker, 8-oxodG and BNIP3 expression [37]. It has also been demonstrated that BNIP3 may act as a mitochondrial sensor of oxidative stress [10]. When activated, BNIP3 changes mitochondrial membrane potential, leading to generation of ROS. In the present study, BNIP3 expression was studied by using specific inhibitors of DNMT and HDACs such as AZA and TSA. AZA-treated cells showed increased expression of BNIP3 in MCF-7 cells. However, TSA-treated cells showed no alteration in the expression of BNIP3. It appears that the increase in BNIP3 expression by Mitocur-1 may modulate the DNA methylation mechanisms. However, the epigenetic regulation of increased expression of BNIP3 by mitocurcuminoids\ requires further investigation. In a recent study, we observed that breast tumors exhibiting hypomethylation of BNIP3 had elevated 8-oxodG compared to tumors exhibiting intermediate to high level of methylation [38]. Differential methylation patterns were reported across different cancer cell lines where in certain cell lines exhibited hypermethylation of BNIP3 associated with its silencing [39], [40]. However, there is no concluding link of this BNIP3 hypermethylation with cytotoxicity. In a recent study, we have shown that HAEC cells lack BNIP3 expression, which was restored upon treatment with 5-aza cytidine concluding that hypermethylation is associated with silencing of BNIP3 [41].

In summary, this study demonstrated that conjugation of curcuminoids with TPP cation, showed significant accumulation in mitochondria in MCF-7 cells. Mitocur-1 induced ROS generation, a drop in ΔØ_m_, and inhibited cell proliferation by inducing cell-cycle arrest leading to apoptotic cell death. Although it was seen that mitocurcuminoid treatment greatly enhanced the generation of ROS, at this time we do not know the mechanisms involved in this process. However, it was observed that mitocurcuminoid-induced generation of ROS and cell death is significantly attenuated in the presence of NAC, thereby suggesting that oxidative stress at least, in part plays a role in mitocurcumiod-mediated cancer cell death.

Further, the inhibition of proliferation was mediated through the inhibition of Akt and STAT3 phosphorylation, and activation of ERK1/2 phosphorylation. Overall, it appears that curcumin can alter several phosphorylation events involved in various cellular processes. This study further highlights two important mechanisms by which Mitocur-1 induces increased expression of BNIP3 namely by (i) induction of ROS and a drop in ΔØm, and (ii) methylation of BNIP3 promoter. Studies utilizing animal models are warranted in order to study the uptake, distribution and efficacy of these mitochondrially-targeted curcuminoids against various animal tumor models.

## Supporting Information

File S1 **A**, Synthesis of Mitocurcuminoids-1, 2 and 3; **B**, Determination of superoxide using EPR spectroscopy **C**, Structural analysis of mitocurcuminoid-1, 2, & 3; **D**, Cellular uptake of mitocurcuminoid-1, 2, 3 or curcumin by MCF-7 cells.
(DOCX)

Table S1 **High Resolution Mass Spectrophotometry (HRMS) data for curcumin, mitocurcuminoid-1, 2, and 3.**
(PDF)

Figure S1 **Synthetic scheme of mitocurcuminoid-1.**
(TIF)

Figure S2 **The ESI-MS spectra of A,** Curcumin; **B,** Mitocurcuminoid-1; **C,** Mitocurcuminoid- 2; **D,** Mitocurcuminoid-3.
(TIF)

Figure S3 **The ESI-MS/MS spectra fragmentation pattern of curcumin and mitocurcuminoids.** **A**, Curcumin (*m/z* 369; CE = 35 eV); **B**, Mitocurcuminoid-1 (*m/z* 487; CE = 35 eV); **C**, Mitocurcuminoid-2 (*m/z* 671; CE = 35 eV); **D**, Mitocurcuminoid3(*m/z*457;CE = 40 eV).
(TIF)

Figure S4 **ESI-MS spectra of curcumin (10 µM) treated MCF -7 cells.** **A**, cytosolic fraction and **B**, mitochondrial fraction. Insert shows the calibration curve of mitocurcuminoid-1 (10 µM) treated MCF-7 cells: cytosolic fraction and mitochondrial fraction. Value corresponding to each point is an average of triplicate measurements made on different days. The error bars in the figure represent the standard deviations from three triplicate measurements.
(TIF)

Figure S5 **ESI-MS spectra of mitocurcuminod-1 (10 µM) treated MCF-7 cells.** **A**, cytosolic fraction and **B**, mitochondrial fraction. . Insert shows the calibration curve of Mitocurcuminoid-2 (10 µM) treated MCF cells: cytosolic fraction and mitochondrial fraction. Value corresponding to each point is an average of triplicate measurements made on different days. The error bars in the figure represent the standard deviations from three triplicate measurements.
(TIF)

Figure S6 **ESI-MS spectra of mitocurcuminoid-2 (10 µM) treated MCF-7 cells.** **A**, cytosolic fraction and **B**, mitochondrial fraction. Insert shows the calibration curve of mitocurcuminoid-3 (10 µM) treated MCF cells: cytosolic fraction and mitochondrial fraction. Value corresponding to each point is an average of triplicate measurements made on different days. The error bars in the figure represent the standard deviations from three triplicate measurements.
(TIF)

Figure S7 **ESI-MS spectra of mitocurcuminoid-3 (10 µM) treated MCF cells.** **A**, cytosolic fraction and **B**, mitochondrial fraction. Insert shows the calibration curve of curcumin (10 µM) treated MCF cells: cytosolic fraction. Value corresponding to each point is an average of triplicate measurements made on different days. The error bars in the figure represent the standard deviations from three triplicate measurements.
(TIF)

Figure S8 **Cell Viability of MCF-10A treated with various concentrations of curcumin and mitocurcuminoids.** MCF-10A cells were incubated with various concentrations of curcumin and mitocurcuminoids-1, 2 and 3 for 24 h. At the end of treatments, cell viability was assayed by SRB assay.
(TIF)

Figure S9 **Cell Viability of MCF-10A treated with various concertrations of triphenylphosphonium (TPP^+^).** MCF-7 cells were incubated with various concentrations of TPP^+^ for 24 h. At the end of treatments, cell viability was assayed by SRB assay.
(TIF)
