# Supplementary material for: Mitochondrial-Targeted Curcuminoids: A Strategy to Enhance Bioavailability and Anticancer Efficacy of Curcumin
Source: PLoS One. 2014 Mar 12;9(3):e89351. doi: 10.1371/journal.pone.0089351 (PMC3951186; doi:10.1371/journal.pone.0089351)
Supplement: File S1 — A, Synthesis of Mitocurcuminoids-1, 2 and 3; B, Determination of superoxide using EPR spectroscopy C, Structural analysis of mitocurcuminoid-1, 2, & 3; D, Cellular uptake of mitocurcuminoid-1, 2, 3 or curcumin by MCF-7 cells. (DOCX) [file pone.0089351.s001.docx]

**SUPPORTING INFORMATION**

**METHODOLOGY:**

**Synthesis of Mitocurcuminoids:**

**1. Mitocurcuminoid-1**

**4-(3-Chloropropoxy)-3-methoxybenzaldehyde**: A mixture of vanillin (5 g, 30.2 mM), 1-bromo-3-chloropropane (10.36 ml, 61.1 mM), potassium carbonate (18.1 g, 122.6 mM) and PEG-400 (15 ml) was stirred at room temperature for 3 h. The mixture was diluted with crushed ice and acidified with diluted HCl. After stirring for 15 min, hexane (100 ml) was added to the solution and stirred for 30 min. The precipitated solid was filtered, washed with hexane and dried (5.1 g, 68%), mp 46–48^o^C. ^1^H NMR (400 MHz, CDCl_3_): δ 9.86 (1H, s), 7.45 (1H, d, *J*=8.4, 2.0 Hz), 7.42 (1H, d, *J*=2.0 Hz), 7.01 (1H, d, *J*=8.4 Hz), 4.26 (2H, t, *J*=6.0 Hz), 3.92 (3H, s), 3.78 (2H, t, *J*=6.2 Hz), 2.33 (2H, quintet, *J*=6.1 Hz).

**(1E,6E)-1,7-Bis[4-(3-chloropropoxy)-3-methoxyphenyl]hepta-1,6-diene-3,5-dione**: To a solution of boric oxide (1.05 g, 15.22 mM) in DMF (4 ml) was added acetyl acetone (1.35 ml, 13.13 mM) followed by tributyl borate (7.16 ml, 26.52 mM) at 65 ^o^C and stirred for 15 min. To the above borate complex, 4(3-chloropropoxy)-3-methoxybenzaldehyde (6 g, 26.25 mM) was added and stirred for 5 min. A mixture of n-butylamine (0.25 ml) and acetic acid (0.75 ml) in DMF (4 ml) was added to the reaction mixture and heated to 95 ^o^C for 4 h. After cooling to 15 ^o^C, acetic acid (20%, 150 ml) was added with stirring and again the reaction mixture was stirred at 70 ^o^C for another 1 h. Then it was cooled to room temperature and extracted with chloroform. The combined chloroform layer was washed with brine and dried over sodium sulfate. The solution was filtered and evaporated the solvent. The residue was chromatographed over silica gel column using hexane-ethyl acetate (80:20) as eluent to give the product as yellow color solid (3.5 g, 26%), mp 132–134^o^C.

**1,7-Bis{3-methoxy-4-[3-(triphenylphosphonium)propoxy]-phenyl}hepta-1,6-diene-3,5-dione dichloride**: A mixture of 1,7-Bis[4-(3-chloropropoxy)-3-methoxyphenyl]hepta-1,6-diene-3,5-dione (350 mg, 0.67 mM), triphenyl phosphine (0.7 g, 2.68 mM), n-butanol (5 ml) and catalytic amount of potassium iodide was stirred at 100 ^o^C for 4 h. The reaction mixture was attained to room temperature and the solution was slowly added with stirring to diethyl ether (75 ml) for 10 min. Few drops of chloroform was added to dissolve the residue in the R.B. and added this solution to the ether. After the addition, the solution was stirred for 30 min and filtered, washed with ether and dried to give the product. The crude product was chromatographed over silica gel column using chloroform-methanol (90:10) as eluents to give the product, which was dissolved in minimum amount of chloroform and slowly added to diethyl ether with stirring. The precipitated product was filtered, washed with ether and dried to give the product as an yellow color solid (450 mg, 65%), mp 130–140^o^C. ^1^H NMR (400 MHz, DMSO-d_6_): δ 2.04 (4H, m), 3.74 (4H, m), 3.86 (6H, s), 4.19 (4H, m), 6.19 (1H, s), 6.89 (2H, d, *J*=15.6 Hz), 7.01 (2H, d, *J*=8.0 Hz), 7.27 (2H, d, *J*=8.0 Hz), 7.39 (2H, br s), 7.61 (2H, d, *J*=15.6 Hz), 7.79–7.94 (30 H, m); ^13^C NMR (100 MHz, DMSO-d_6_): δ 183.2, 149.6, 149.4, 140.2, 134.9 (d, *J*=3 Hz), 133.6 (d, *J*=10 Hz), 130.3 (d, *J*=12 Hz), 128.3, 122.6, 122.5, 118.3 (d, *J*=86 Hz), 113.5, 111.1, 101.0, 67.7 (d, *J*=17 Hz), 55.9, 22.2, 17.8 (d, *J*=53 Hz); HPLC: 96.5%.

**2. Mitocurcuminoid-2**

**(1E,6E)-1-[4-(3-chloropropoxy)-3-methoxyphenyl]-7-(4 hydroxy-3 methoxyphenyl)hepta-1,6-diene-3,5-dione**: To a solution of boric oxide (1.75 g, 25.4 mM) in DMF (5 ml) was added acetyl acetone (2.24 ml, 21.9 mM) followed by tributyl borate (11.7 ml, 44.27 mM) at 65 ^o^C and stirred for 15 min. To the above borate complex, 4(3-chloropropoxy)-3-methoxybenzaldehyde (5 g, 21.9 mM), vanillin (2.67 g, 21.9 mM) were added and stirred for 5 min. A mixture of n-butylamine (0.38 ml) and acetic acid (1.26 ml) in DMF (5 ml) was added to the reaction mixture and heated to 95 ^o^C for 4 h. After cooling to 15 ^o^C, acetic acid (20%, 175 ml) was added with stirring and again the reaction mixture was stirred at 70 ^o^C for another 1 h. Then it was cooled to 5-10^o^C, filtered the solids, washed with ice cold water and dried. The crude product was chromatographed over silica gel column using hexane-ethyl acetate (90:10) as eluent to give the product as yellow color solid (750 mg, 12%), mp 160–164^o^C.

**1-{3-methoxy-4-[3-(triphenylphosphonium)propoxy]phenyl}-7[(3-methoxy-4 hydroxy)phenyl]hepta-1,6-diene-3,5-dione chloride**: A mixture of 1-[4-(3-chloropropoxy)-3-methoxyphenyl]-7-(4-hydroxy-3-methoxyphenyl)hepta-1,6-diene-3,5-dione (550 mg, 1.23 mM), triphenyl phosphine (0.64 g, 2.4 mM), n-butanol (5 ml) and catalytic amount of potassium iodide was stirred at 100 ^o^C for 4 h. The reaction mixture was attained to room temperature and the solution was slowly added with stirring to diethyl ether (75 ml) for 10 min. Few drops of chloroform was added to dissolve the residue in the R.B. and added this solution to the ether. After the addition, the solution was stirred for 30 min and filtered, washed with ether and dried to give the product. The crude product was chromatographed over silica gel column using chloroform-methanol (90:10) as eluents to give the product, which was dissolved in minimum amount of chloroform and slowly added to diethyl ether with stirring. The precipitated product was filtered, washed with ether and dried to give the product as an yellow color solid (560 mg, 64%), mp 130–140^o^C. ^1^H NMR (400 MHz, DMSO-d_6_): δ 2.03 (2H, m), 3.73 (2H, t, *J*=14.4 Hz), 3.85 (6H, s), 4.18 (2H, t, *J*=5.6 Hz), 6.13 (1H, s), 6.78 (1H, d, *J*=15.6 Hz), 6.84 (1H, d, *J*=8.0 Hz), 6.86 (1H, d, *J*=16.0 Hz), 7.00 (1H, d, *J*=8.4 Hz), 7.17 (1H, d, *J*=8.4 Hz), 7.25 (1H, d, *J*=8.0 Hz), 7.34 (1H, s), 7.38 (1H, s), 7.58 (1H, d, *J*=15.6 Hz), 7.59 (1H, d, *J*=16.0 Hz), 7.78–7.94 (15 H, m), 9.65 (1H, s); ^13^C NMR (100 MHz, DMSO-d_6_): δ 183.8, 182.5, 149.5, 149.4, 148.0, 140.9, 139.9, 134.9 (d, *J*=3 Hz), 133.6 (d, *J*=10 Hz), 130.3 (d, *J*=12 Hz), 128.4, 126.3, 123.1, 122.6, 122.5, 121.2, 118.3 (d, *J*=86 Hz), 115.8, 113.5, 111.6, 111.0, 100.8, 67.7 (d, *J*=17 Hz), 55.9, 55.8, 22.2, 17.8 (d, *J*=52 Hz); LC-MS (positive scan): *m/z* 671 (M–Cl)^+^; HPLC: 99.1%.

**3.Mitocurcuminoid-3**

**1,7-Bis[4-(3-chloropropoxy)phenyl]hepta-1,6-diene-3,5-dione**: To a solution of boric oxide (2.01 g, 29.23 mM) in DMF (5.3 ml) was added acetyl acetone (2.5 ml, 25.1 mM) followed by tributyl borate (13.4 ml, 50.9 mM) at 65 ^o^C and stirred for 15 min. To the above borate complex, 4(3-chloropropoxy)benzaldehyde (10 g, 50.4 mM) was added and stirred for 5 min. A mixture of n-butylamine (0.44 ml) and acetic acid (1.45 ml) in DMF (5.3 ml) was added to the reaction mixture and heated to 95 ^o^C for 4 h. After cooling to 15 ^o^C, acetic acid (20%, 350 ml) was added with stirring and again the reaction mixture was stirred at 70 ^o^C for another 1 h. Then it was cooled to 5-10^o^C, filtered the solids, washed with ice cold water and dried. The residue was chromatographed over silica gel column using hexane-ethyl acetate (80:20) as eluent to give the product as yellow color solid (7.5 g, 32%).

**1,7-{4-[3-(triphenylphosphonium)propoxy]phenyl}hepta-1,6-diene-3,5-dione dichloride**: A mixture of 1,7-Bis[4-(3-chloropropoxy)phenyl]hepta-1,6-diene-3,5-dione (3.0 g, 6.5 mM), triphenyl phosphine (6.81 g, 26 mM), n-butanol (25 ml) and catalytic amount of potassium iodide was stirred at 100 ^o^C for 8 h. The reaction mixture was attained to room temperature and the solution was slowly added with stirring to diethyl ether (75 ml) for 10 min. Few drops of chloroform was added to dissolve the residue in the R.B. and added this solution to the ether. After the addition, the solution was stirred for 30 min and filtered, washed with ether and dried to give the product. The crude product was chromatographed over silica gel column using chloroform-methanol (90:10) as eluents to give the product, which was dissolved in minimum amount of chloroform and slowly added to diethyl ether with stirring. This process was repeated for 4 times. The precipitated product was filtered, washed with ether and dried to give the product as an yellow color solid (2.2 g, 34%), mp 145–160^o^C. ^1^H NMR (400 MHz, DMSO-d_6_): δ 2.03 (4H, m), 3.78 (4H, m), 4.21 (4H, m), 6.17 (1H, s), 6.82 (2H, d, *J*=15.6 Hz), 7.02 (4H, d, *J*=8.4 Hz), 7.62 (2H, d, *J*=15.6 Hz), 7.70 (4H, d, *J*=8.0 Hz), 7.79–7.92 (30 H, m); ^13^C NMR (100 MHz, DMSO-d_6_): δ 183.2, 159.8, 139.9, 134.9 (d, *J*=3 Hz), 133.6 (d, *J*=10 Hz), 130.3 (d, *J*=13 Hz), 130.1, 127.7, 122.1, 118.3 (d, *J*=86 Hz), 115.1, 101.2, 66.9 (d, *J*=17 Hz), 22.1, 17.9 (d, *J*=52 Hz); HPLC: 94.4%.

**Determination of superoxide using EPR spectroscopy**

Electron paramagnetic resonance (EPR) spectroscopy was used to determine the superoxide-production capability by mitcurcuminoid-1 or curcumin. MCF-7 cells (4×10^6^ cells) were incubated with curcumin (10 μM), mitocurcuminoid-1 (10 μM), or mitocurcuminoid-1 (10 μM), + MnTBAP (Mn(III)tetrakis(4-benzoic acid), porphyrin chloride (10 μM) in 6 ml of medium containing glucose (1 M), CaCl_2_ (200 mM), DTPA (5.9 mM), NaCl (4.54 M), and KCl (370 mM) in sodium phosphate buffer (2.35 M NaH_2_PO_4_ and 7.61 M Na_2_HPO_4_ at pH 7.4) with 40 mM of spin trap 5,5-dimethyl-pyrroline N-oxide (DMPO), incubated at 37^o^ C for 20 min. In a separate experiment, superoxide generating system consisting of xanthine (0.5 mM) + xanthine oxidase (0.1 U/ml) and DMPO 40 mM in PBS were added and EPR measurements were performed. EPR conditions: Microwave frequency, 9.786 GHz; microwave power, 10 mW; modulation amplitude, 1 G; scan time, 30 s; no. of scans, 10.

**RESULTS:**

**Structural analysis of mitocurcuminoid-1, 2, & 3**

The mitocurcuminoid-1, 2, 3 and curcumin (Fig. 1) were analyzed under positive ion ESI-MS conditions and spectra are shown in Supplementary information, Fig. S2. The curcumin showed [M+H]^+^ ion at m/z 369. The mitocurcuminoid-1, 2, 3 were ionic (preformed ions), as expected the positive ion ESI spectra showed the positive ion part of the molecule. The mitocurcuminoid-1 and 3 showed [M]^2+^ ion at m/z 487 and 457, respectively, and the mitocurcuminoid-2 showed [M]^+^ ion at m/z 671. The high-resolution mass spectrometry (HRMS) data of these ions confirmed their elemental compositions (Supplementary information Table. S1). Further MS/MS experiments were performed for the above-detected ions (Supplementary information Fig. S3) and the spectra provided the structural information of the selected ions.

**Cellular uptake of mitocurcuminoid-1, 2, 3 or curcumin by MCF-7 cells**

The mitochondrial and cytosolic extracts from untreated MCF cells were considered as the blank samples. The ESI spectra of the blank samples showed a set of ions that include a few common ions (m/z 122, 136, 365 and 707). The ions pertinent to the target molecules were absent in the blank spectra. The spectra of all cytosolic fractions from treated cells include the ion corresponding to the respective target compound that was treated with, in addition to the matrix peaks as obtained in the case of the blank sample. Similarly, the spectra of all mitochondrial fractions from treated cells, except curcumin-treated cells, showed the presence of respective target compound. The relative abundances of the peaks due to mitocurcuminoids are always more in the mitochondrial fraction than in the cytosolic fraction (Supplementary information Fig. S4-S7). The MS/MS spectra of the ions of target molecules from the respective treated cells matched well with those of the standard. It was found that mitocurcuminoids-1, 2, and 3 accumulated in mitochondria but the untagged curcumin was not detected in the mitochondrial fraction. The MS analysis also indicated that, after intracellular and intramitochondrial accumulation, TPP-tagged curcuminoids remain intact without undergoing hydrolysis to TPP and curcumin.

**Table S1: High Resolution Mass Spectrophotometry data for curcumin, mitocurcuminoid-1, 2, and 3.**

| Compound | Observed  ion | Molecular  Formula | Theoretical  mass (*m/z*) | Measured  mass (*m/z*) | Error  (ppm) |
| --- | --- | --- | --- | --- | --- |
| Curcumin | [M+H]^+^ | C_21_H_21_O_6_ | 369.1338 | 369.1351 | 3.49 |
| Mitocur-1 | [M]^2+^ | C_63_H_60_O_6_P_2_ | 487.1927 | 487.1915 | -2.48 |
| Mitocur-2 | [M]^+^ | C_42_H_40_O_6_P | 671.2562 | 671.2556 | -0.97 |
| Mitocur-3 | [M]^2+^ | C_61_H_56_O_4_P_2_ | 457.1821 | 457.1817 | -0.97 |
