# Supplementary material for: Mitochondrial-Targeted Curcuminoids: A Strategy to Enhance Bioavailability and Anticancer Efficacy of Curcumin
Source: PLoS One. 2014 Mar 12;9(3):e89351. doi: 10.1371/journal.pone.0089351 (PMC3951186; doi:10.1371/journal.pone.0089351)
Supplement: Table S1 — High Resolution Mass Spectrophotometry (HRMS) data for curcumin, mitocurcuminoid-1, 2, and 3. (PDF) [file pone.0089351.s002.pdf]

Table S1: High Resolution Mass Spectrophotometry data for curcumin, mitocurcuminoid-1, 2, and 3.

Compound	Observed ion	Molecular Formula	Theoretical mass (*m/z*)	Measured mass (*m/z*)	Error (ppm)
Curcumin	$[M+H]^+$	$C_{21}H_{21}O_6$	369.1338	369.1351	3.49
Mitocur-1	$[M]^{2+}$	$C_{63}H_{60}O_6P_2$	487.1927	487.1915	-2.48
Mitocur-2	$[M]^+$	$C_{42}H_{40}O_6P$	671.2562	671.2556	-0.97
Mitocur-3	$[M]^{2+}$	$C_{61}H_{56}O_4P_2$	457.1821	457.1817	-0.97
