# Supplementary figures and images for: Mitochondrial-Targeted Curcuminoids: A Strategy to Enhance Bioavailability and Anticancer Efficacy of Curcumin
Source: PLoS One. 2014 Mar 12;9(3):e89351. doi: 10.1371/journal.pone.0089351 (PMC3951186; doi:10.1371/journal.pone.0089351)

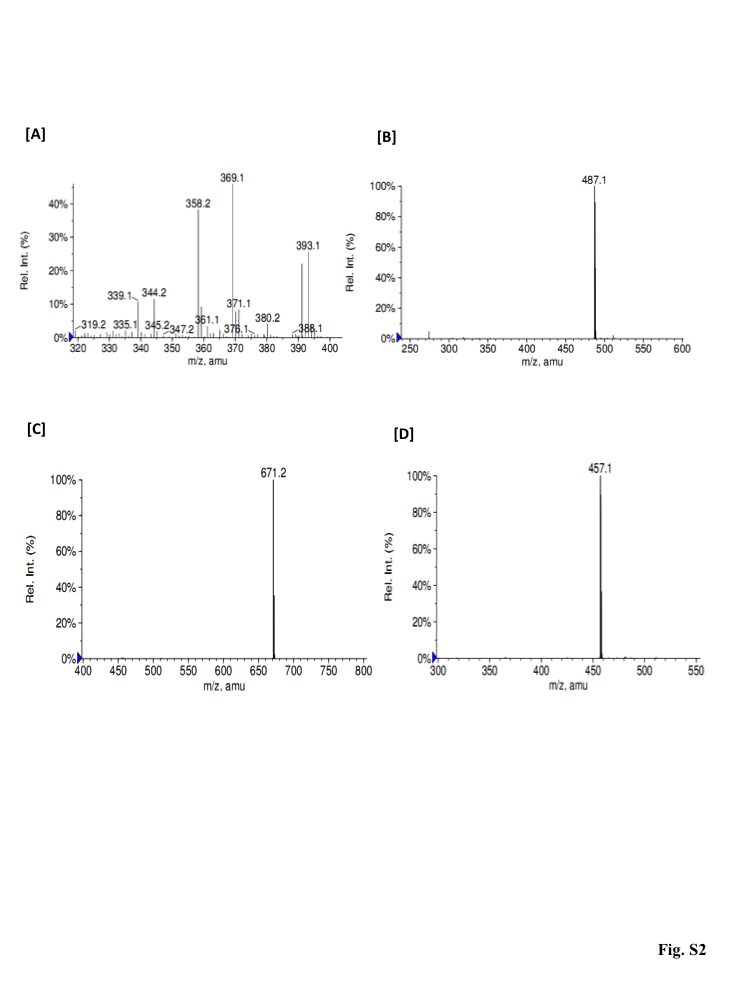

Supplement: Figure S2 — The ESI-MS spectra of A, Curcumin; B, Mitocurcuminoid-1; C, Mitocurcuminoid- 2; D, Mitocurcuminoid-3. (TIF) [file pone.0089351.s004.tif]

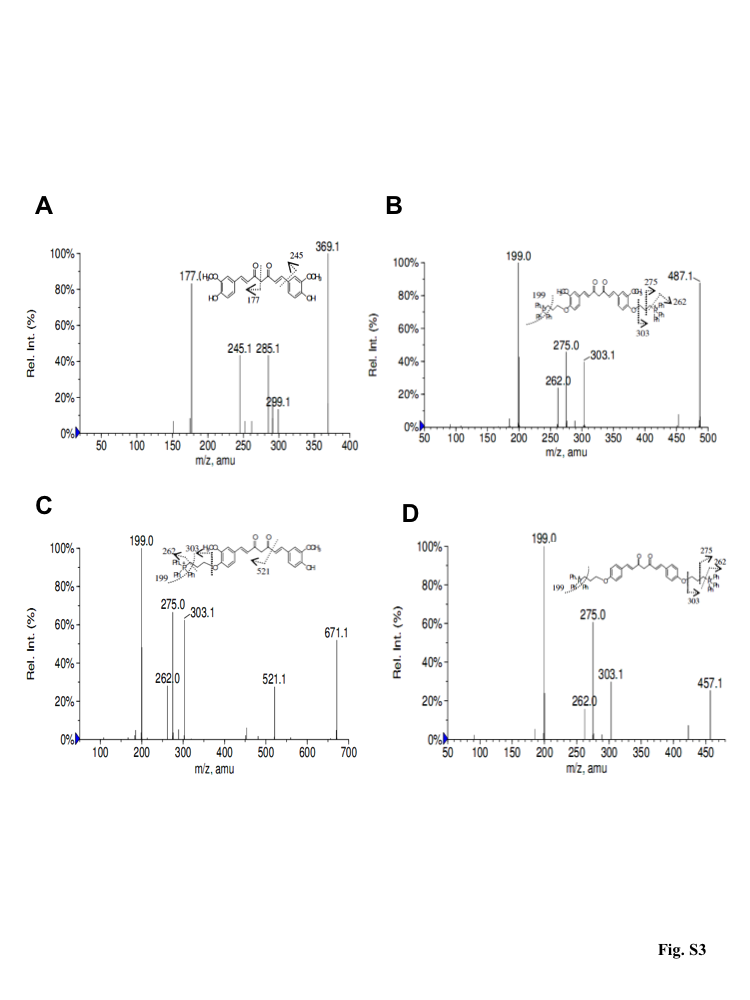

Supplement: Figure S3 — The ESI-MS/MS spectra fragmentation pattern of curcumin and mitocurcuminoids. A, Curcumin (m/z 369; CE = 35 eV); B, Mitocurcuminoid-1 (m/z 487; CE = 35 eV); C, Mitocurcuminoid-2 (m/z 671; CE = 35 eV); D, Mitocurcuminoid3(m/z457;CE = 40 eV). (TIF) [file pone.0089351.s005.tif]

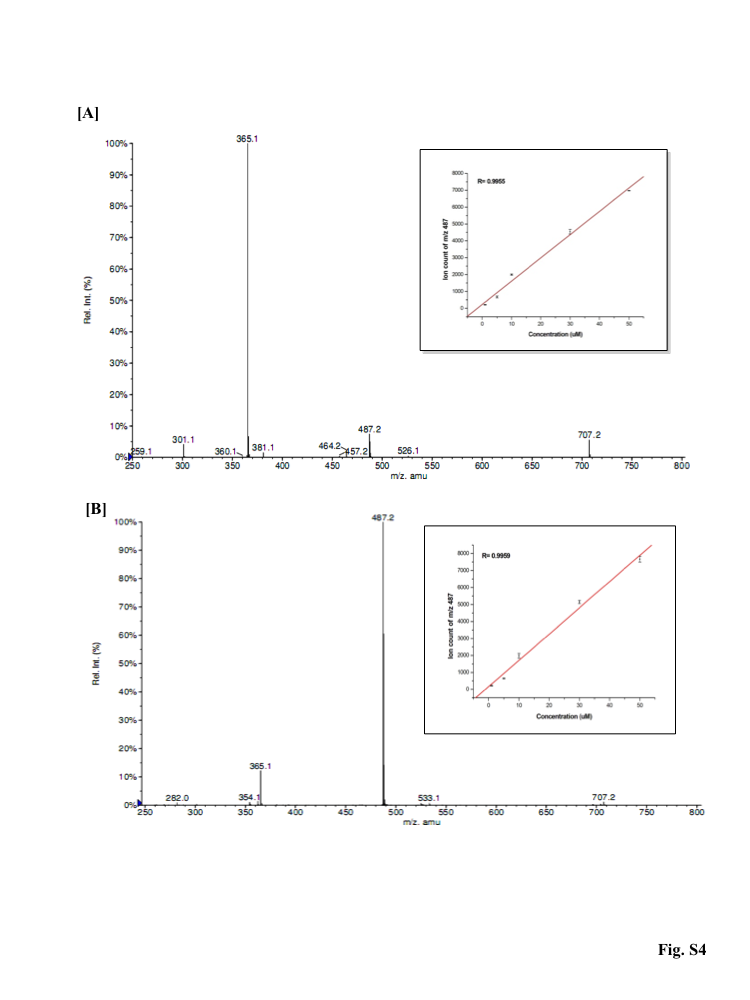

Supplement: Figure S4 — ESI-MS spectra of curcumin (10 µM) treated MCF -7 cells. A, cytosolic fraction and B, mitochondrial fraction. Insert shows the calibration curve of mitocurcuminoid-1 (10 µM) treated MCF-7 cells: cytosolic fraction and mitochondrial fraction. Value corresponding to each point is an average of triplicate measurements made on different days. The error bars in the figure represent the standard deviations from three triplicate measurements. (TIF) [file pone.0089351.s006.tif]

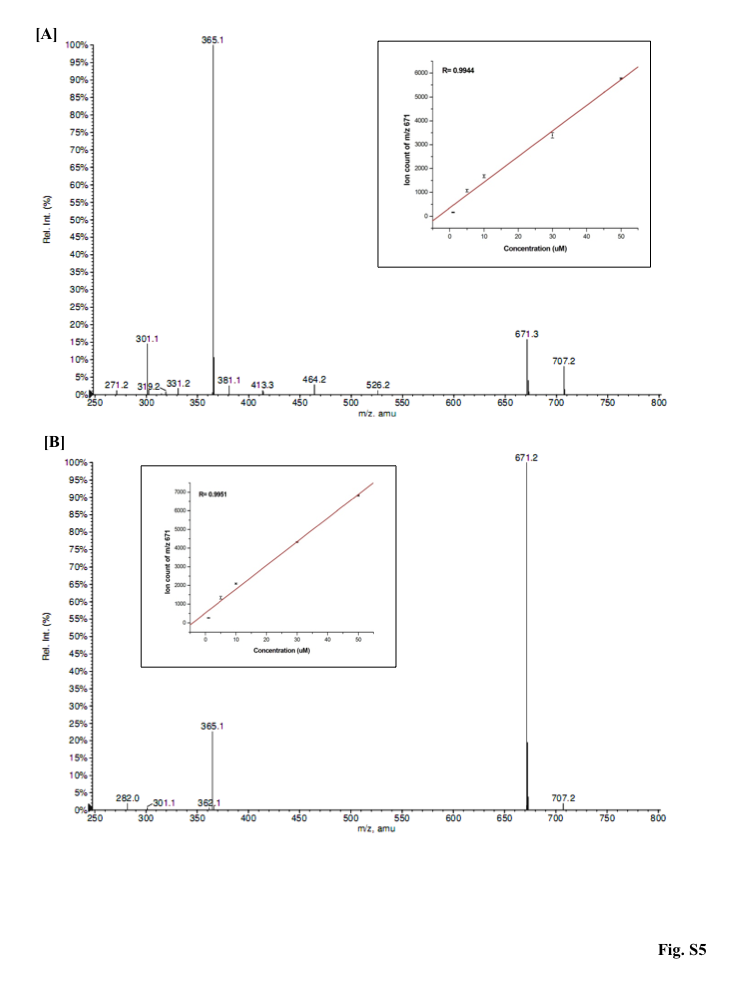

Supplement: Figure S5 — ESI-MS spectra of mitocurcuminod-1 (10 µM) treated MCF-7 cells. A, cytosolic fraction and B, mitochondrial fraction. . Insert shows the calibration curve of Mitocurcuminoid-2 (10 µM) treated MCF cells: cytosolic fraction and mitochondrial fraction. Value corresponding to each point is an average of triplicate measurements made on different days. The error bars in the figure represent the standard deviations from three triplicate measurements. (TIF) [file pone.0089351.s007.tif]

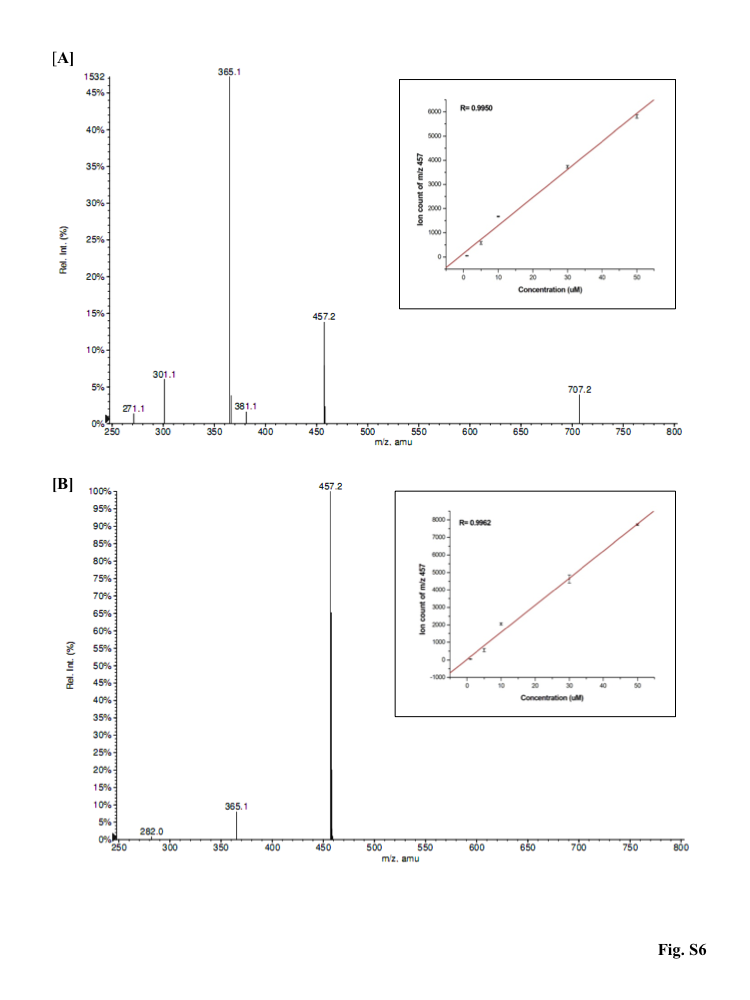

Supplement: Figure S6 — ESI-MS spectra of mitocurcuminoid-2 (10 µM) treated MCF-7 cells. A, cytosolic fraction and B, mitochondrial fraction. Insert shows the calibration curve of mitocurcuminoid-3 (10 µM) treated MCF cells: cytosolic fraction and mitochondrial fraction. Value corresponding to each point is an average of triplicate measurements made on different days. The error bars in the figure represent the standard deviations from three triplicate measurements. (TIF) [file pone.0089351.s008.tif]
